# Novel loci for flag leaf thickness with breeding potential: genetic dissection and candidate genes prediction

**DOI:** 10.3389/fpls.2026.1906045

**Published:** 2026-07-29

**Authors:** Jiajun Liu, Lei Tao, Nana Qin, Jingtao Liu, Defu Wang, Xiaorong Liu, Yuyin Li, Hongbin Peng, Li Zhang, Jixuan Heng, Xingyu Chen, Jian Ma

**Affiliations:** 1 Dazhou Key Laboratory of Agricultural Resources Development and Ecological Conservation in Daba Mountain/School of Ecotourism, Sichuan University of Arts and Science, Dazhou, China; 2 Variety Experiment Department, Sichuan Seed Management Station, Chengdu, China; 3 Cereal and Oil Crops Research Institute, Dazhou Academy of Agricultural Sciences, Dazhou, China; 4 Coarse Grains Research Institute, Chongqing Three Gorges Academy of Agricultural Sciences, Chongqing, China; 5 State Key Laboratory of Crop Gene Exploration and Utilization in Southwest China/Triticeae Research Institute, Sichuan Agricultural University, Chengdu, China

**Keywords:** wheat, flag leaf thickness, quantitative trait locus (QTL), candidate genes, breeding potential

## Abstract

As a key morphological trait of flag leaves, flag leaf thickness (FLT) directly modulates light energy capture efficiency and per-unit-area photosynthetic capacity, and is thus identified as a critical regulator of wheat grain yield formation. Dissecting the genetic basis underlying FLT is of great significance for accelerating the molecular breeding of high-yield wheat varieties. In this study, two recombinant inbred line (RIL) populations were employed to map quantitative trait locus (QTL) for FLT across five independent environments. A total of 19 QTLs controlling FLT were identified in the two RIL populations. Among them, *QFLT.suas-2CN-4B.2*, *QFLT.suas-2CN-6A*, *QFLT.suas-2SY-3B.2*, and *QFLT.suas-2SY-6A* exhibited stable expression across multiple environments, and comparative analysis with previously reported QTLs indicated that all four loci are likely novel. Notably, *QFLT.suas-2CN-6A* and *QFLT.suas-2SY-6A* were co-localized within the same physical interval, suggesting they are likely the same locus. Candidate gene analysis for the co-localized locus *QFLT.suas-2CN-6A* and *QFLT.suas-2SY-6A* identified seven putative candidate genes highly expressed in wheat leaves, all of which encode chlorophyll a-b binding proteins that may participate in the regulation of FLT development. Furthermore, significant positive correlations between FLT and spikelet number per spike (SNS) were detected in both RIL populations. The favorable alleles of *QFLT.suas-2CN-6A* and *QFLT.suas-2SY-6A* significantly increased SNS by 3.21% and 3.31%, respectively. Collectively, these results deepen our understanding of the genetic basis underlying wheat FLT, and provide stable, valuable QTL resources and candidate gene targets for molecular marker-assisted breeding of high-yield wheat.

## 1 Introduction

Common wheat (*Triticum aestivum* L.) is one of the most widely cultivated staple cereal crops worldwide, feeding approximately one-third of the global population (Ali et al., 2022; Bhurta et al., 2022). Therefore, sustaining high and stable wheat yields is a core priority for global food security.

As the uppermost functional leaf in wheat, the flag leaf serves as the primary source of photosynthetic assimilates transported to developing grains after heading (Siddiqui et al., 2021; Zanella et al., 2023). Flag leaf-related traits, including flag leaf length (FLL), flag leaf width (FLW), flag leaf area (FLA), and flag leaf thickness (FLT), are core determinants of wheat plant architecture and yield formation. The global leaf economics spectrum theory demonstrates that thicker leaves, as a key component of the resource-acquisitive strategy, typically have higher leaf nitrogen content and greater photosynthetic capacity (Wright et al., 2004; Onoda et al., 2017). Specifically, FLT regulates both the amount of light absorbed by leaf tissue and the diffusion efficiency of CO_2_ within the mesophyll, directly affecting photosynthetic rate (Agustí et al., 1994; Syvertsen et al., 1995). Therefore, dissecting the genetic basis of FLT is of great significance for improving photosynthetic efficiency and grain yield in wheat.

To date, quantitative trait loci (QTLs) for FLL, FLW, and FLA have been identified across almost all 21 wheat chromosomes, providing abundant genetic resources for molecular breeding of flag leaf morphology (Ma et al., 2020; Niu et al., 2023, 2025; Tu et al., 2021). Moreover, many flag leaf-related genes have been cloned, such as *TaWAK2-A* (Du et al., 2024), *RG1* (Bai et al., 2024) and *WPA1* (Chen et al., 2024). Although increased leaf thickness has been proven to enhance grain yield by improving photosynthetic efficiency and optimizing plant architecture in other cereal crops (Li et al., 2009; Wang et al., 2026), the genetic control of FLT in wheat remains poorly understood.

Currently, only a limited number of QTL mapping studies for wheat FLT have been reported. For instance, Wang et al. (2022) detected two major stable QTLs, *QFLT-2B* and *QFLT-6A*, across three environments (including the BLUP dataset), which explained 7.18%–8.11% and 6.77%–8.09% of the phenotypic variance (PVE), respectively. Xu et al. (2024) identified two additional FLT QTLs, *Qflt.saw-2B* and *Qflt.saw-3B*, with PVE ranging from 5.78% to 13.30% and 6.29% to 12.32%, respectively. Niu et al. (2025) further mapped four FLT QTLs (*Qflt-3D.1*, *Qflt-3D.2*, *Qflt-4A*, and *Qflt-5A*) with closely linked molecular marker. Overall, the number of major, stably expressed FLT QTL remains very limited, and most of the reported loci have relatively small phenotypic effects, which cannot meet the needs of molecular breeding for high-yield wheat ideotypes.

QTL pleiotropism, where a single locus simultaneously controls two or more traits, offers significant theoretical and practical value in the genetic analysis of complex traits in wheat. It is especially prevalent in wheat, owing to large genome size of wheat and the continuous variation of most agronomic traits (Zhao et al., 2025; Hui et al., 2023). For example, the pleiotropic QTL *QFll.sicau-2D* can simultaneously increase FLL, spike length, spikelet number per spike (SNS), grain number per spike, and grain length (Liu et al., 2018b). The SNP marker *AX-111015470* on chromosome 1A was associated with dough stability time, extension area and maximum resistance in wheat, suggesting this locus was pleiotropic (Zhao et al., 2024).

Given the critical role of FLT in wheat yield formation and the scarcity of identified major QTL associated with FLT, it is urgent to explore diverse wheat germplasm resources to identify novel, stably expressed major loci controlling this trait. In this study, we performed QTL mapping for FLT used two recombinant inbred line (RIL) populations based on high-density genetic maps constructed previously according to the proposed genetic analysis model (“multiple environmental assessments-depth analysis of individual traits-comprehensive evaluation of various traits-friendly marker development-verification in different backgrounds”) (Zhang et al., 2024). The main objectives were: (1) to identify major and stable QTL for FLT across multiple environments; (2) to analyze the correlation between FLT and yield-related traits, and the effect of the major FLT QTL on yield-related traits; (3) to detect FLT QTL independent of other flag leaf traits via conditional QTL analysis; and (4) to predict candidate genes for the major FLT QTL. These results will provide a theoretical basis for molecular marker-based selection of wheat lines with optimal FLT, and facilitate the genetic improvement of wheat yield through plant architecture optimization.

## 2 Materials and methods

### 2.1 Plant materials

Two previously reported RIL populations developed using the single-seed descent method were used for QTL mapping in this study. They included 20828/Chuannong 16 (CN16) F_7_ RILs (2CN, 199 lines) (Liu et al., 2018a) and 20828/SY95–71 F_7_ RILs (2SY, 126 lines) (Liu et al., 2020). The wheat line 20828 was bred in Chengdu Institute of Biology, Chinese Academy of Sciences characterized by excellent agronomic traits including large flag leaf (Ma et al., 2020; Tu et al., 2021), high SNS (Ma et al., 2019a), suitable plant height (Li et al., 2020) and a high level of resistance to stripe rust (Ma et al., 2019b). CN16 was a national approved cultivar with more productive tiller number (PTN) (Liu et al., 2018a) and high thousand grain weight (TGW) (Ma et al., 2019c). SY95–71 possesses the characteristics of an ideal plant type, which include more tillers (Liu et al., 2020), longer spike extension length (Li et al., 2020), smaller flag leaf angle (Tu et al., 2021) and well-developed root system (Chen et al., 2022). Compared with CN16 and SY95-71, 20828 exhibited thicker FLT (**Table 1**). The 2CN and 2SY populations were obtained from the Triticeae Research Institute at Sichuan Agricultural University.

**Table 1:** Phenotypic variation of flag leaf thickness (FLT) in 2CN and 2SY populations grown in different environments.

Environments	2CN parents		2CN RILs				2SY parents		2SY RILs			
	20828	CN16	Range	Mean ± SD	CV	H2	20828	SY95-71	Range	Mean	CV	H2
2025MC	0.35	0.29 *	0.23–0.40	0.30 ± 0.03	10.18%	0.61	0.34	0.27 *	0.24–0.38	0.31 ± 0.03	10.45%	0.47
2025BD	0.37	0.30 **	0.27–0.46	0.36 ± 0.04	10.19%		0.43	–	0.22–0.43	0.33 ± 0.05	14.26%	
2025PZ	0.27	0.21 *	0.17–0.36	0.24 ± 0.03	12.49%		0.28	0.24 *	0.16–0.36	0.25 ± 0.03	11.78%	
2026BD	0.34	0.26 **	0.19–0.36	0.28 ± 0.03	9.28%		0.31	0.26 **	0.21–0.35	0.27 ± 0.02	8.16%	
2026KJ	0.29	0.24 **	0.18–0.35	0.25 ± 0.03	11.44%		0.28	0.21 *	0.14–0.34	0.26 ± 0.03	11.27%	
BLUP	0.32	0.27	0.24–0.34	0.29 ± 0.02	5.37%		0.31	0.27	0.26–0.31	0.29 ± 0.01	4.20%	

RILs, recombinant inbred lines; CN16, chuannong 16; SD, standard deviation; *CV*, coefficient of variation; *H^2^*, broad-sense heritability; BLUP, best linear unbiased prediction; * and ** represent significant differences at the 0.05 and 0.01 levels; - represents data missing.

### 2.2 Phenotypic evaluation

The 2CN and 2SY populations were planted at Beidou (BD, 107°45′E, 31°15′N), Micheng (MC, 107°19′E, 31°18′N), Pengzhou (PZ, 103°58′E, 30°59′N) and Kaijiang (KJ, 107°50′E, 31°13′N) in 2024–2026. In each environment, the population was planted under a randomized complete block design with a single block. All field trials were conducted using a single-row planting design with a row length of 1.5 m, 15 seeds per row, plant spacing of 0.1 m, and row spacing of 0.3 m. Plots were independently randomized within each environment. Standard local agronomic management practices were applied throughout the growing season. FLT phenotypes of the two populations were evaluated across five environments: 2025BD, 2026BD, 2025MC, 2025PZ and 2026KJ. According to the method of previous research (Wang et al., 2026), a leaf thickness measurement machine (TP-S05A, https://www.tpynkj.com/) with a measurement accuracy of 0.001 mm was used for measuring FLT in the 2CN and 2SY populations. The measurement machine was recalibrated after measuring the FLT of each line. We measured the thickness of the flag leaf middle section after the wheat flowering stage. For each line, three flag leaves with similar growth conditions were randomly selected from the main tillers of individual plants.

Additionally, the best linear unbiased prediction (BLUP) datasets of other traits in 2CN and 2SY populations were obtained from our previously published studies, including: PTN (Liu et al., 2018a, 2020), SNS (Ding et al., 2022; Ma et al., 2019a), FLL, FLW and FLA (Ma et al., 2020; Tu et al., 2021), and TGW (Ma et al., 2019c; Qu et al., 2021).

### 2.3 QTL mapping

Two genetic maps constructed previously (Liu et al., 2018a, 2020) were used for QTL mapping in this study. The inclusive composite interval mapping (ICIM) of the biparental population module (BIP) in IciMapping v4.2 (Meng et al., 2015) was used to detect QTL for FLT in the 2CN and 2SY populations. The QTL walking speed was set at 1.0 cM with the PIN value of 0.001. Based on 1000 permutations of BIP module in QTL IciMapping, the logarithm of odds (LOD) threshold was set as 3.53 in 2CN population and 3.51 in 2SY population. The function of multi-environmental trials (MET-ADD) of QTL IciMapping was used to identify interaction between QTL and environments (Step = 1 cM, PIN = 0.001, and LOD = 3.53 in 2CN population and 3.51 in 2SY population). Epistatic interactions were identified using the epistatic function (EPI) in QTL IciMapping (LOD = 3.53 in 2CN population and 3.51 in 2SY population, PIN = 0.001, and step = 1.0 cM). A QTL is classified as a major QTL when its average PVE greater than 10%, and as a stable QTL when it is consistently detected across multiple environments. QTL was named based on the QTL was named based on the guidelines for gene nomenclature in wheat (Boden et al., 2023). ‘suas’ represents Sichuan University of Arts and Science.

### 2.4 Conditional QTL analysis

Conditional QTL analysis is commonly used to dissect the independent genetic effects of a target trait excluding the interference of its correlated traits (Cui et al., 2011; Li et al., 2020; Wang et al., 2025). Conditional QTL analysis was performed using QGAStation 2.0 and QTL IciMapping v4.2 to enhance QTL mapping accuracy and mitigate potential confounding effects from linked traits. QGAStation 2.0 (Chen et al., 2012) was used to calculated the conditional phenotypic values (FLT|FLL, FLT|FLW, and FLT|FLA). QTL IciMapping v4.2 was also used to detect the conditional QTL for conditional phenotypic values with pre-adjusted parameters: Step = 1.0 cM, PIN = 0.001, and LOD = 2.50. Compared with the LOD value obtained from unconditional QTL, if the LOD value from conditional QTL decreases or increases but remains above 2.5, the QTL is considered to be partially correlated with other traits without having an independent effect. Conversely, if the LOD value shows no significant change, the QTL effect is considered to be independent of other traits.

### 2.5 Comparison with previously reported QTL and prediction of candidate genes

The sequences of the flanking markers linked with the major QTL in this study were derived from our previous study (Liu et al., 2018a, 2020). The physical position of the major QTL was determined via sequence alignment of its flanking markers against the Chinese Spring reference genome (CS v2.1 genome) (Zhu et al., 2021). The annotations and expression patterns for predicted genes were searched on WheatOmics (https://wheatomics.sdau.edu.cn//).

To confirm whether the presently identified QTL is a novel locus, we compared physical intervals of the previously reported QTL or genes related to FLT by anchoring their sequences of flanking markers on genome assembly of CS v2.1 genome.

### 2.6 Data analysis

The SPSS Statistic 24 program (IBM, Armonk, NY, USA; https://www.ibm.com/products/spss-statistics) was used for conducting the independent sample *t*-test, and calculating the Pearson correlation coefficient. Additionally, SAS v8.0 (SAS Institute, Cary, NC, USA; https://www.sas.com/) was used to analyze the best linear unbiased prediction (BLUP) and broad-sense heritability (*H^2^*) of the FLT among the investigated environments. Analysis of variance (ANOVA) of multi-environment trials was performed using QTL IciMapping v4.2 to detect interaction between genotypes and environments. Origin Pro 2021 v9.8.3 (https://www.originlab.com/) was used to draw figures.

## 3 Result

### 3.1 Phenotypic analyses

In 2CN population, the FLT of the parents 20828 and CN16 ranged from 0.27 mm to 0.37 mm and from 0.21 mm to 0.30 mm, respectively (**Table 1**). The FLT of the parents 20828 and SY95–71 ranged from 0.28 mm to 0.43 mm and from 0.21 mm to 0.27 mm in 2SY population, respectively (**Table 1**). 20828 had significantly higher FLT value than CN16 and SY95-71 (**Table 1**). The FLT ranged from 0.17 mm to 0.46 mm in the 2CN population and from 0.14 mm to 0.43 mm in the 2SY population (**Table 1**). The coefficients of variation (*CV*) of the 2CN and 2SY populations ranged from 5.13% to 12.49% and from 4.03% to 14.26%, respectively. The *H^2^* of the 2CN and 2SY populations were 0.61 and 0.47 (**Table 1**), respectively, indicating FLT was controlled by both genetic and environmental factors.

In addition, the frequency distribution of the FLT in 2CN and 2SY populations was continuous distributions approximating normality, suggesting that FLT are typical quantitative trait (**Supplementary Figure 1**). Significant and positive correlations (*P* < 0.05) for FLT were detected among five environments (except 2026KJ and 2025BD, 2026KJ and 2025PZ in 2SY population) and BLUP dataset (**Supplementary Table 1**). ANOVA revealed that genotype (G), environment (E), and G×E interaction had a significant effect on FLT in the two populations (**Table 2**). Environmental factors (65.52%) were the major contributors to FLT variation in the 2CN population, while both environmental and genetic factors (43.48% and 34.78%) jointly affected FLT in the 2SY population.

**Table 2:** Analysis of variance for flag leaf thickness (FLT) in the two populations.

Populations	Source	Degrees of freedom	Sum of squares	Mean square	F-value	P-value	Estimated variance
2CN RILs	Block/Environment	10	0.0112	0.0011	2.0402	0.02627	0.0000
	Genotype (G)	196	1.3367	0.0068	12.4216	0.00000	0.0005 (17.24%)
	Environment (E)	4	4.1974	1.0493	1911.3032	0.00000	0.0019 (65.52%)
	G×E interaction	703	1.3603	0.0019	3.5244	0.00000	0.0005 (17.24%)
2SY RILs	Block/Environment	10	0.0109	0.0011	1.4515	0.15260	0.0000
	Genotype (G)	127	0.8573	0.0068	8.9705	0.00000	0.0005 (21.74%)
	Environment (E)	4	1.2881	0.3220	427.9020	0.00000	0.0010 (43.48%)
	G×E interaction	407	1.0568	0.0026	3.4504	0.00000	0.0008 (34.78%)

### 3.2 Correlation analysis

**Figure 1:**
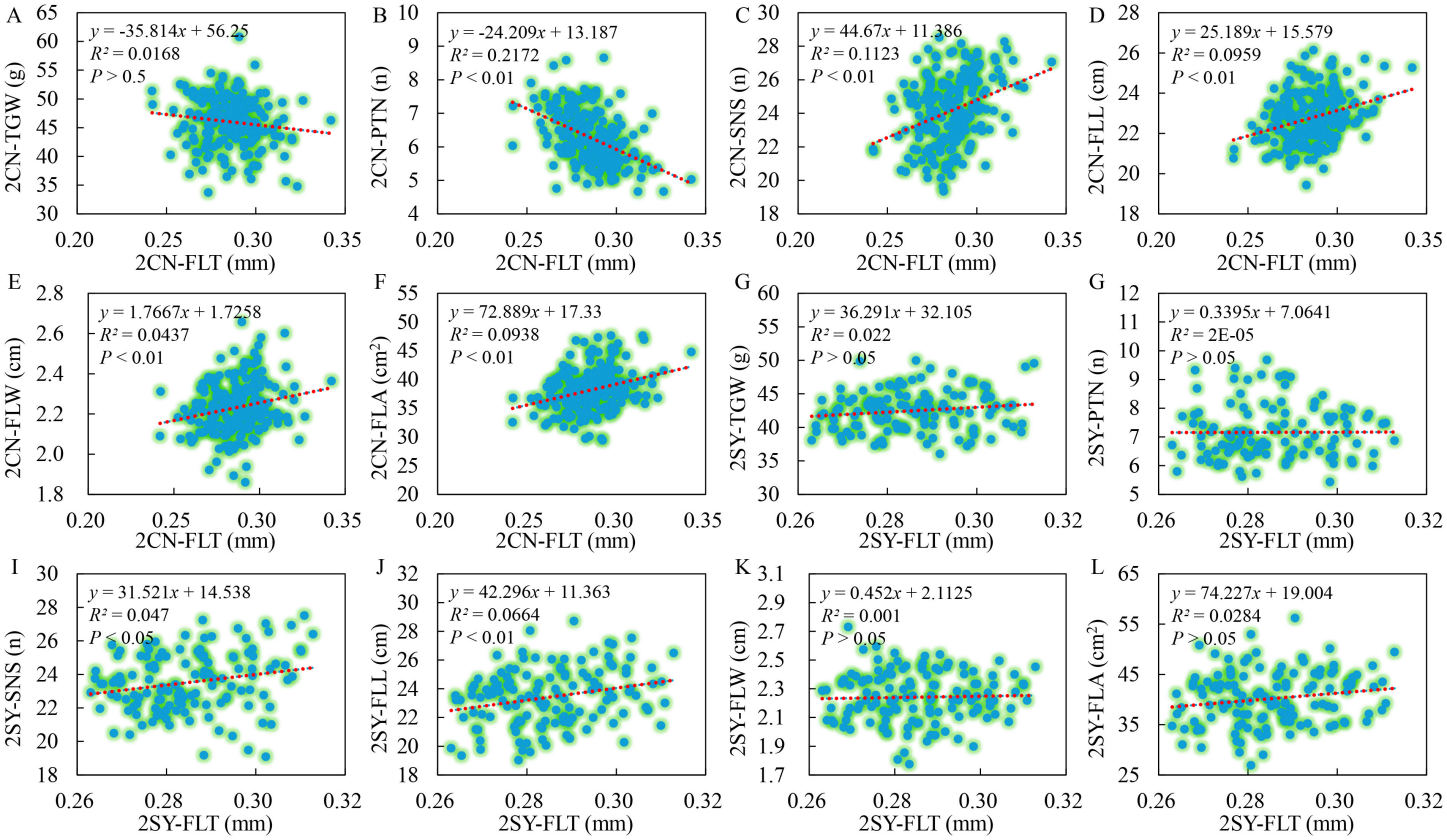
Correlation analysis between flag leaf thickness (FLT) and the yield traits and flag leaf traits. **(A–F)** Correlation analysis in 2CN population; **(G–L)** Correlation analysis in 2SY population. TGW, thousand grain weight; PTN, productive tiller number; SNS, spikelet number per spike; FLL, flag leaf length; FLW, flag leaf width; FLA, flag leaf area.

We also employed the BLUP dataset to systematically analyze the correlations between FLT and other agronomic traits, including TGW, PTN, SNS, FLL, FLW and FLA. Significant and positive correlations (*P* < 0.01) between FLT and SNS, FLL, FLW and FLA (**Figures 1C–F**), significant and negative correlations (*P* < 0.01) between FLT and PTN (**Figure 1B**) and no significant correlations between FLT and TGW (**Figure 1A**) were observed in 2CN population. For 2SY population, FLT showed the significant and positive correlations (*P* < 0.05 or *P* < 0.01) with SNS and FLL (**Figures 1I, J**), whereas no significant correlations were observed between FLT and TGW, PTN, FLW and FLA (**Figures 1G, H, K–L**).

### 3.3 QTL mapping for FLT

Eight and four QTLs for FLT were detected in 2CN and 2SY populations, respectively (**Table 3**). They were located on chromosomes 3A, 3B, 3D, 4B, 4D, 5A, 5D and 6A. Of the 12 QTLs, *QFLT.suas-2CN-4B.2*, *QFLT.suas-2CN-6A*, *QFLT.suas-2SY-3B.2* and *QFLT.suas-2SY-6A* were detected in at least three environments (including the BLUP dataset). The average PVE of these four QTLs were all greater than 10%. Thus, these four QTLs were regarded as the stable and major QTLs. The remaining 8 QTLs were detected in one or two environments and explained the PVE of 3.60%–15.21%, suggesting that they were minor QTLs (**Table 3**). The positive alleles of these four major and stable QTLs were all contributed by the parent 20828 (**Table 3**).

**Table 3:** Quantitative trait loci (QTL) for flag leaf thickness (FLT) identified from different environments in 2CN and 2SY populations.

Population	*QTL*	Env	Chr	Position	*Left marker*	*Right marker*	LOD	PVE (%)	Add	CI
2CN RILs	QFLT.suas-2CN-3A	2026KJ	3A	92	AX-111541125	AX-110455859	4.36	10.25	0.010	91.5–93.5
		BLUP	3A	92	AX-111541125	AX-110455859	3.62	5.61	0.004	
	QFLT.suas-2CN-3D	BLUP	3D	6	AX-111916664	AX-109884133	3.75	4.02	-0.003	4.5–7.5
	QFLT.suas-2CN-4B.1	2026BD	4B	43	AX-111068079	AX-111738217	6.58	15.21	0.010	40.5–46.5
	QFLT.suas-2CN-4B.2	2025MC	4B	48	AX-109526283	AX-110549715	9.09	16.05	0.013	47.5–48.5
		2025BD	4B	48	AX-109526283	AX-110549715	4.80	9.99	0.011	
		2025PZ	4B	48	AX-109526283	AX-110549715	3.82	8.75	0.009	
		2026KJ	4B	48	AX-109526283	AX-110549715	8.66	14.69	0.012	
		BLUP	4B	48	AX-109526283	AX-110549715	16.27	19.95	0.007	
	QFLT.suas-2CN-4D	BLUP	4D	8	AX-109963158	AX-111564466	3.62	3.86	-0.003	6.5–9.5
	QFLT.suas-2CN-5A.1	BLUP	5A	17	AX-111643204	AX-109959535	4.60	5.08	0.004	16.5–18.5
	QFLT.suas-2CN-5A.2	2025BD	5A	25	AX-109916689	AX-110434983	3.94	7.96	0.010	24.5–26.5
	QFLT.suas-2CN-6A	2025BD	6A	62	AX-109868726	AX-109283360	5.57	11.93	0.012	61.5–66.5
		2025MC	6A	63	AX-109283360	AX-110442638	3.69	6.08	0.008	
		BLUP	6A	63	AX-109283360	AX-110442638	11.25	13.02	0.006	
		2026BD	6A	64	AX-109283360	AX-110442638	5.40	11.96	0.009	
2SY RILs	QFLT.suas-2SY-3B.1	BLUP	3B	66	AX-110001564	AX-108757678	4.16	8.20	0.004	65.5–66.5
	QFLT.suas-2SY-3B.2	2025MC	3B	68	AX-111011119	AX-110918765	3.73	15.66	0.013	67.5–68.5
		2026KJ	3B	68	AX-111011119	AX-110918765	5.37	25.46	0.015	
		BLUP	3B	68	AX-111011119	AX-110918765	4.12	8.66	0.004	
	QFLT.suas-2SY-5D	2025MC	5D	117	AX-110405892	AX-89331886	3.82	13.41	0.012	104.5–125.5
	QFLT.suas-2SY-6A	2026BD	6A	65	AX-108870565	AX-110637693	5.57	22.63	0.011	63.5–75.5
		BLUP	6A	70	AX-86175025	AX-109325100	15.50	34.51	0.007	
		2025MC	6A	71	AX-86175025	AX-109325100	5.10	14.94	0.013	
		2025BD	6A	71	AX-86175025	AX-109325100	5.78	10.72	0.023	
		2025PZ	6A	74	AX-109536927	AX-110038163	7.26	26.65	0.016	

RILs, recombinant inbred lines; Env, Environment; Chr, Chromosome; LOD, logarithm of odds; PVE, phenotypic variance explained; Add, additive effect of a QTL; CI, Confidence interval; BLUP, best linear unbiased prediction.

**Figure 2:**
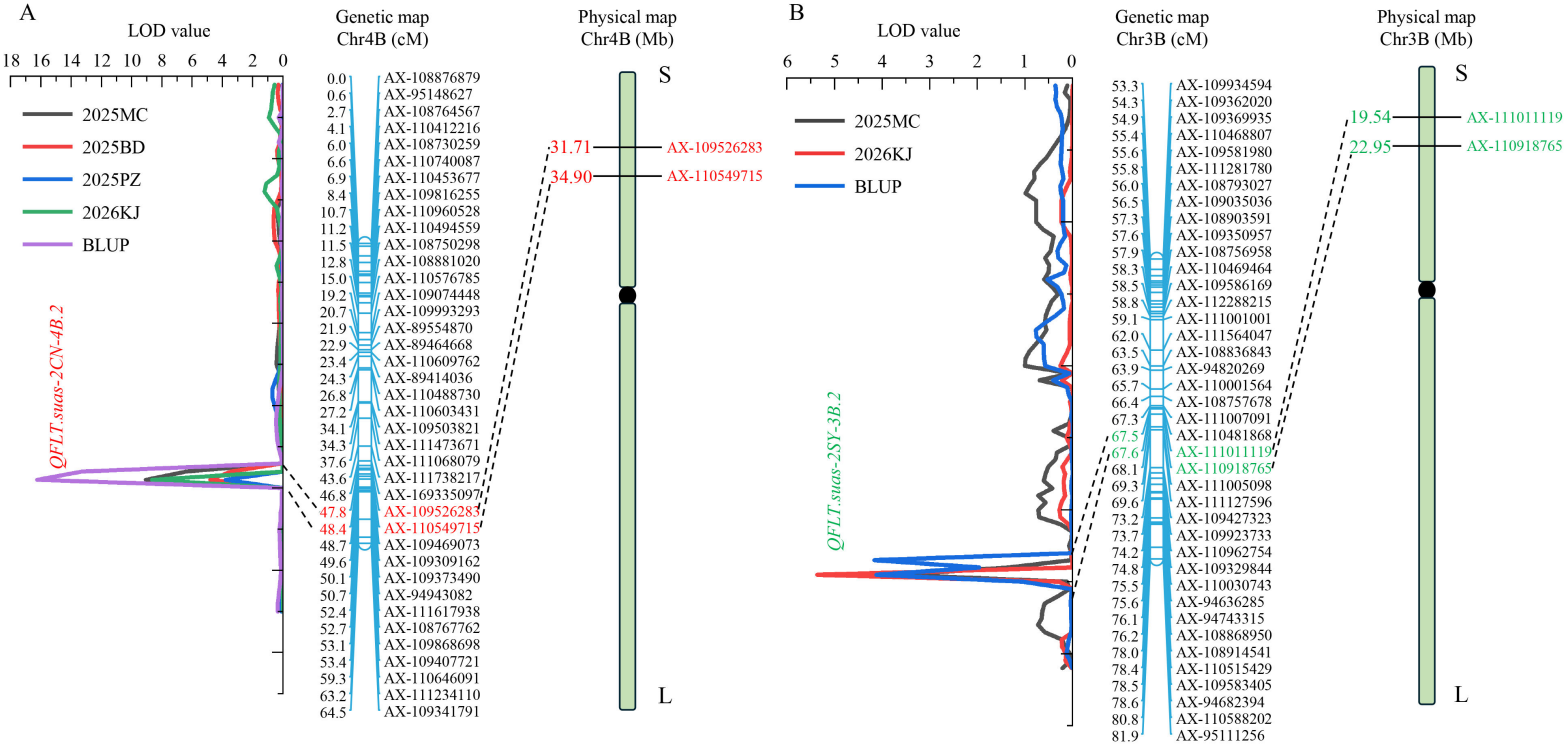
Partial genetic and physical maps of *QFLT.suas-2CN-4B.2* **(A)** and *QFLT.suas-2SY-3B.2* **(B)**. S and L represent chromosome short and long arm, respectively.

**Figure 3:**
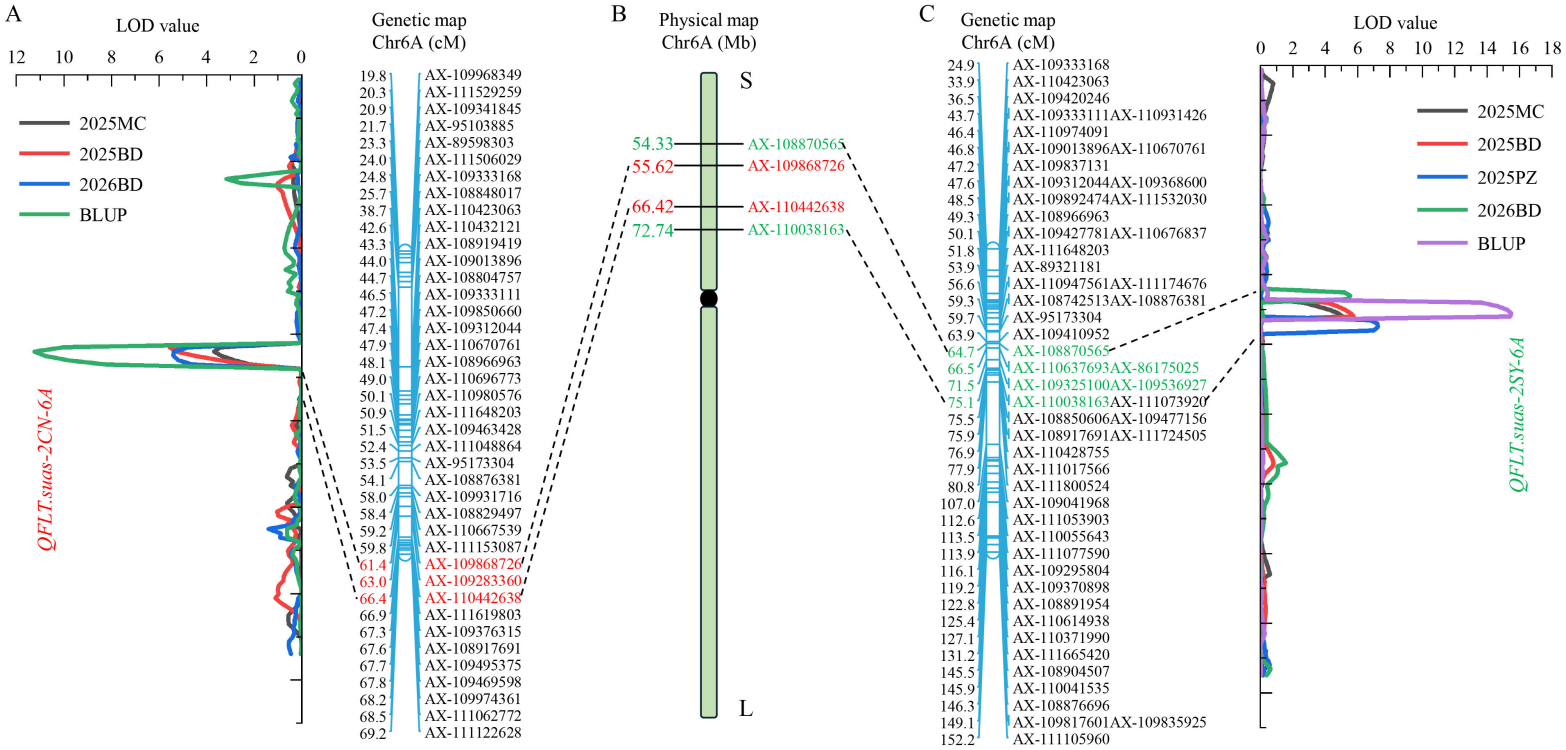
Partial genetic and physical maps of *QFLT.suas-2CN-6A* **(A, B)** and *QFLT.suas-2SY-6A* **(B, C)**. S and L represent chromosome short and long arm, respectively.

In 2CN population, *QFLT.suas-2CN-4B.2*, located on chromosome 4B between markers *AX-109526283* and *AX-110549715*, was identified in four environments and the BLUP dataset, and explained the PVE of 8.75%–19.95% with a LOD value of 3.82–16.27 (**Table 3**, **Figure 2A**). *QFLT.suas-2CN-6A*, detected in three environments as well as the BLUP dataset, was positioned on chromosome 6A between markers *AX-109868726* and *AX-110442638* (**Table 3**, **Figure 3A**). It had a LOD value of 3.69–11.25, accounting for the PVE of 6.08% and 13.02%.

Two stable and major QTLs, *QFLT.suas-2SY-3B.2* and *QFLT.suas-2SY-6A*, were also detected in 2SY population (**Table 3**, **Figures 2B**, **3C**). *QFLT.suas-2SY-3B.2* and *QFLT.suas-2SY-6A* were identified in three and four environments (including the BLUP dataset), respectively. *QFLT.suas-2SY-3B.2* was mapped between markers *AX-111011119* and *AX-110918765* on chromosome 3B, accounting for 8.66%–25.46% of the PVE with a LOD value of 3.73–5.37. *QFLT.suas-2SY-6A* with a LOD value of 5.57–15.50 was located on chromosome 6A between markers *AX-108870565* and *AX-110038163*, explaining 10.72%–34.51% of the PVE.

Analysis results of the multi-environment QTL analysis are listed in **Supplementary Table 2**. A total of 29 QTLs were detected in 2CN and 2SY populations. Most of them had low LOD and PVE values. *QFLT.suas-2CN-4B.2*, *QFLT.suas-2CN-6A*, *QFLT.suas-2SY-3B.2* and *QFLT.suas-2SY-6A* were simultaneously detected by multi-environmental and individual environmental analyses, providing further evidence that they are major and stable loci.

### 3.4 Physical intervals of the four major QTLs

The physical intervals of the four QTLs were obtained by anchoring the sequences of their flanking markers to CS v2.1 genome. *QFLT.suas-2CN-4B.2* and *QFLT.suas-2CN-6A* detected in the 2CN population were mapped between 31.71 Mb and 34.90 Mb on chromosome arm 4BS and between 55.62 Mb and 66.42 Mb on chromosome arm 6AS, respectively (**Figures 2A**, **3A, B**). *QFLT.suas-2SY-3B.2* and *QFLT.suas-2SY-6A* identified in the 2SY population were located between 19.54 Mb and 22.95 Mb on chromosome 3B and between 54.33 Mb and 72.74 Mb on chromosome arm 6AS, respectively (**Figures 2B**, **3B, C**). Interestingly, an overlapping physical interval of 10.80 Mb was observed between *QFLT.suas-2CN-6A* and *QFLT.suas-2SY-6A*, suggesting that these two QTLs were likely the same genetic loci, supporting the consistency of the identified locus.

### 3.5 Conditional QTL analysis between FLT and other flag leaf-related traits

**Figure 4:**
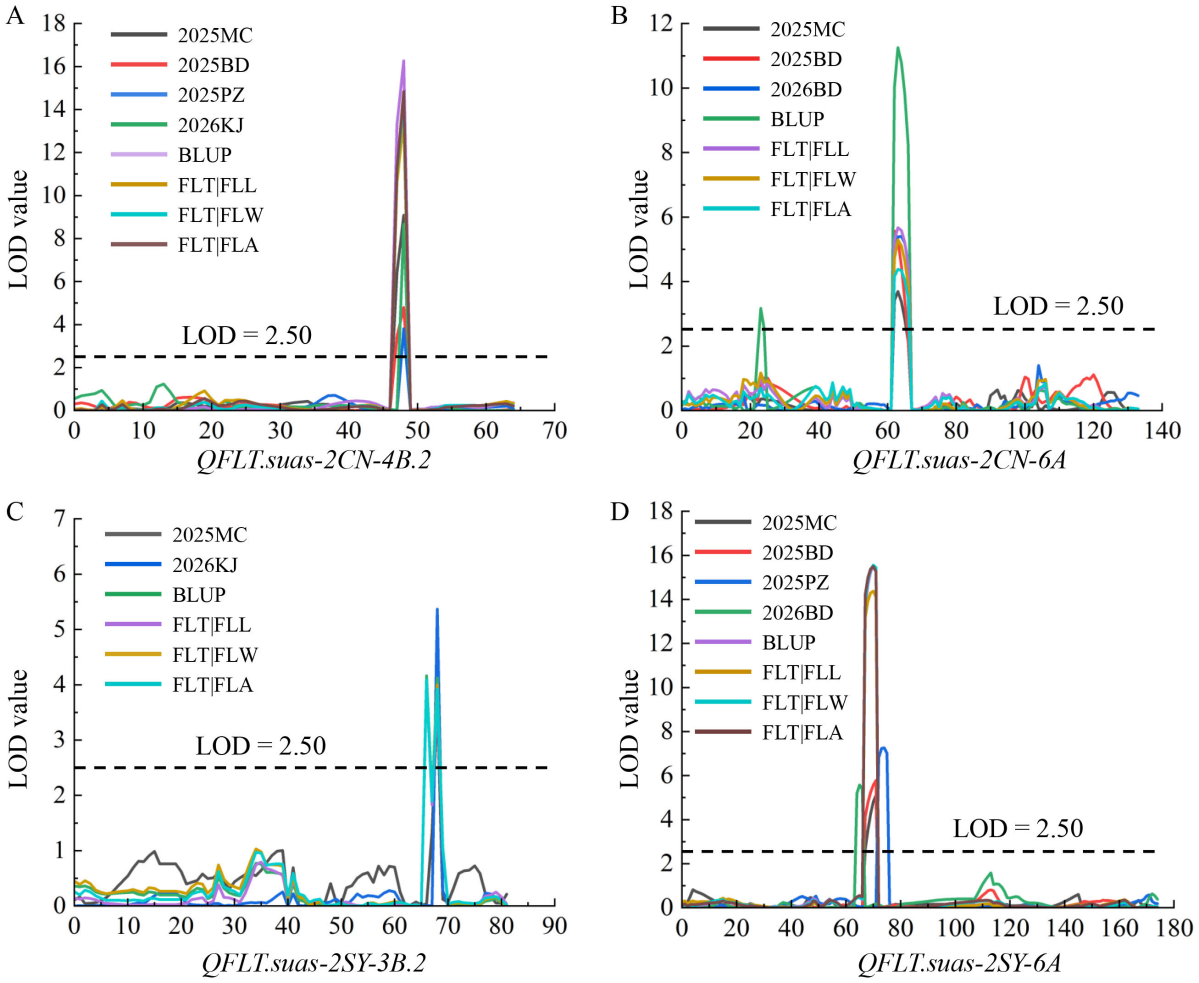
Logarithm of odds (LOD) values of conditional QTL for flag leaf thickness (FLT). **(A)** *QFLT.suas-2CN-4B.2*; **(B)** *QFLT.suas-2CN-6A*; **(C)** *QFLT.suas-2SY-3B.2*; **(D)** *QFLT.suas-2SY-6A*.

FLL, FLW, FLA and FLT are all important component of flag leaf morphology. Conditional QTL analysis was carried out to dissect the genetic relationship between FLT and other flag leaf-related traits (FLL, FLW and FLA). When FLL, FLW and FLA were used as conditioning factors, the four identified QTLs (*QFLT.suas-2CN-4B.2*, *QFLT.suas-2CN-6A*, *QFLT.suas-2SY-3B.2* and *QFLT.suas-2SY-6A*) were consistently located in their respective target genomic intervals (**Figure 4**; **Supplementary Table 3**). No obvious differences were observed for the LOD value of *QFLT.suas-2CN-4B.2*, *QFLT.suas-2SY-3B.2* and *QFLT.suas-2SY-6A* when FLT was conditional on FLL, FLW and FLA (**Figures 4A, C, D**). However, the LOD value of *QFLT.suas-2CN-6A* obviously decreased when FLT was conditional on FLL, FLW and FLA (**Figure 4B**). These results suggested that *QFLT.suas-2CN-6A* were closely related to FLL, FLW and FLA, while *QFLT.suas-2CN-4B.2*, *QFLT.suas-2SY-3B.2* and *QFLT.suas-2SY-6A* were independent of FLL, FLW and FLA.

### 3.6 Effects of four major QTLs on FLT

**Figure 5:**
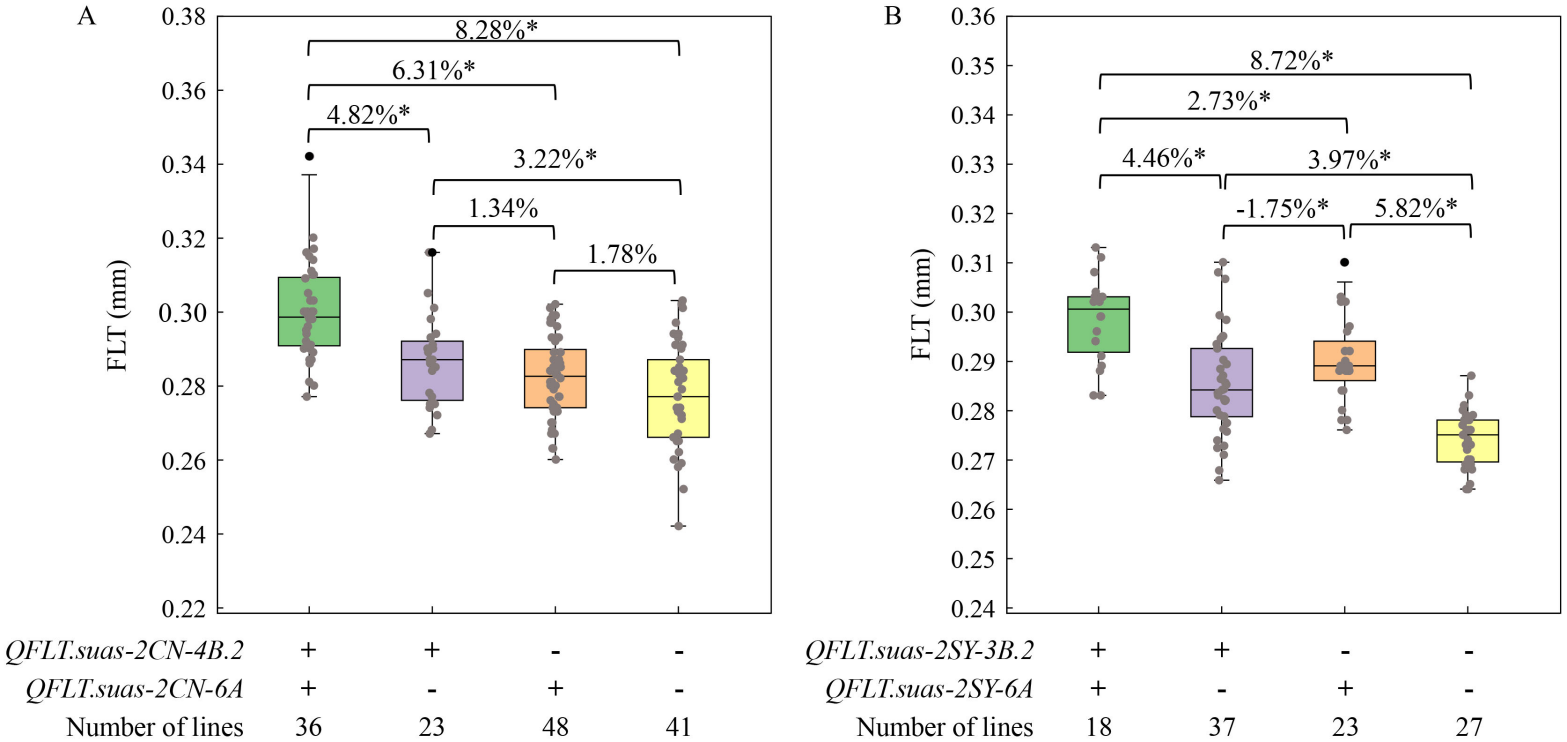
Effects of four major quantitative trait loci (QTLs) for flag leaf thickness (FLT) in the 2CN and 2SY populations. **(A)** Effects of *QFLT.suas-2CN-4B.2* and *QFLT.suas-2CN-6A* for FLT in the 2CN population; **(B)** Effects of *QFLT.suas-2SY-3B.2* and *QFLT.suas-2SY-6A* for FLT in the 2SY population. + and - represent lines with and without the positive alleles of the target QTL, respectively. *represents significant differences at the 0.05 levels.

The positive alleles of *QFLT.suas-2CN-4B.2*, *QFLT.suas-2CN-6A*, *QFLT.suas-2SY-3B.2* and *QFLT.suas-2SY-6A* were derived from the parent 20828 (**Table 3**). The effects of these alleles on FLT were further analyzed in the 2CN and 2SY populations (**Figure 5**).

In 2CN population, compared with RILs without any of the alleles increasing FLT, RILs only possessing the positive allele from *QFLT.suas-2CN-4B.2* significantly increased FLT by 3.22%, those only possessing the positive allele from *QFLT.suas-2CN-6A* increased FLT by 1.78%, and those with the combination of *QFLT.suas-2CN-4B.2* and *QFLT.suas-2CN-6A* significantly increased FLT by 8.28% (**Figure 5A**). RILs with the combination of positive alleles of *QFLT.suas-2CN-4B.2* and *QFLT.suas-2CN-6A* significantly increased FLT by 4.82% and 6.31%, respectively, compared to those with the positive allele of either *QFLT.suas-2CN-4B.2* or *QFLT.suas-2CN-6A* (**Figure 5A**).

In the 2SY population, compared with RILs carrying no FLT-increasing alleles, RILs only harboring the positive allele from *QFLT.suas-2SY-3B.2* significantly increased FLT by 3.97%, those only harboring the positive allele from *QFLT.suas-2SY-6A* significantly increased FLT by 5.82%, and those with the combination of *QFLT.suas-2SY-3B.2* and *QFLT.suas-2SY-6A* significantly increased FLT by 8.72% (**Figure 5B**). Compared to RILs with the positive allele of either *QFLT.suas-2SY-3B.2* or *QFLT.suas-2SY-6A*, those with the combination of positive alleles of *QFLT.suas-2SY-3B.2* and *QFLT.suas-2SY-6A* significantly increased FLT by 4.46% and 2.73%, respectively (**Figure 5B**).

### 3.7 Effects of *QFLT.suas-2CN-6A* and *QFLT.suas-2SY-6A* on yield-related traits

The effects of the major loci, *QFLT.suas-2CN-6A* and *QFLT.suas-2SY-6A*, on yield-related traits were analyzed (**Supplementary Figure 2**). Lines carrying the positive allele of *QFLT.suas-2CN-6A* in the 2CN population exhibited a decrease of -1.42% in TGW and -0.74% in PTN, but a significant increase of 3.21% in SNS (**Supplementary Figures 2A–C**). In the 2SY population, lines carrying the positive allele of *QFLT.suas-2SY-6A* exhibited an increase of 0.23% in TGW, 2.46% in PTN, and a significant increase of 3.31% in SNS (**Supplementary Figures 2D–F**). These findings demonstrate that these two loci were associated with increased SNS.

## 4 Discussion

### 4.1 FLT is of great significance to gain yield

FLT is a core anatomical trait closely linked to the mesophyll structure, chloroplast density, leaf anatomical configuration, and photosynthetic efficiency (Terashima et al., 2010), forming a coordinated morphological physiological regulatory network that determines the carbon assimilation capacity in wheat. Thick flag leaves typically exhibit a tighter mesophyll cell arrangement, with increased cell layers, enlarged palisade tissue, and reduced intercellular air space (Kalve et al., 2014). This optimized mesophyll structure provides more attachment sites for chloroplasts and significantly increases chloroplast density per unit leaf area. In contrast, thin flag leaves usually possess loose mesophyll tissue and sparse chloroplast distribution, which limits light capture and CO_2_ fixation. Collectively, these differences in leaf anatomical architecture directly shape the photosynthetic performance of the flag leaves.

Flag leaf morphological traits are key determinants of final grain yield and biomass accumulation (Liu et al., 2015). Optimizing flag leaf architectural traits is widely recognized as a viable pathway for unlocking wheat yield potential in modern breeding programs (Kumar et al., 2024). However, solely increasing the FLA has inherent constraints, such as excessive FLA, which intensifies canopy shading, reduces the light utilization efficiency of the population, and ultimately limits grain yield. Against this backdrop, FLT, a critical morphological index independent of leaf area, provides a solution to this trade-off. FLT directly modulates leaf light energy capture efficiency and photosynthetic capacity per unit area (Xu et al., 2024), thereby governing grain yield formation without the adverse effects associated with an enlarged FLA. Four QTLs controlling FLT were detected in this study (**Table 3**). Among them, *QFLT.suas-2CN-4B.2*, *QFLT.suas-2SY-3B.2* and *QFLT.suas-2SY-6A* were independent of FLL, FLW and FLA (**Figures 4A, C, D**). *QFLT.suas-2CN-6A* and *QFLT.suas-2SY-6A* significantly increased the SNS by 3.21% and 3.31%, respectively (**Supplementary Figures 2C, F**). These results show that FLT is a highly promising target trait for breeding high-yield wheat and provides a key entry point for subsequent genetic dissection and molecular marker-assisted selection.

### 4.2 The inconsistent correlation between FLT and other agronomic traits in two RIL populations

The inconsistent correlation between FLT and other agronomic traits across different mapping populations, which likely reflects the influence of genetic background on trait relationships on complex trait relationships. In this study, we observed divergent correlation patterns between the two RIL populations. In the 2CN population, FLT exhibited a significant negative correlation with PTN (*P* < 0.01), and significant positive correlations with FLW and FLA (*P* < 0.01, **Figures 1B, E, F**). By contrast, no significant correlations between FLT and PTN, FLW and FLA were observed in the 2SY population (**Figures 1H, K, L**). These population-specific correlation patterns are further supported by previously published studies in wheat germplasm with distinct genetic backgrounds. For example, in the double haploid (DH) population derived from the cross between Jinchun 7 and Jinmai 919, FLT showed no correlations with FLL, FLW, FLA and TGW (Wang et al., 2022). In the RIL population developed from DH118/Jinmai 919, Xu et al. (2024) found that FLT was significantly positively correlated with SNS, TGW and FLW, significantly negatively correlated with FLA, and showed no significant correlation with FLL. More recently, Niu et al. (2025) reported significant positive correlations between FLT and FLW, FLA and TGW, but no significant correlation with FLL in another DH population derived from Baiqimai/Neixiang 5. Taken together, the correlations between FLT and other agronomic traits are not a fixed relationship, but is strongly modulated by population-specific genetic background factors.

### 4.3 Influence of genetic background on FLT QTL detection

The detection of QTL is affected by the genetic background of the mapping population as well as by complicated epistatic interactions. In this study, eight and four QTLs were detected in the 2CN and 2SY populations, respectively, which shared a common parent (**Table 3**), but only one same major QTL located on chromosome 6A was identified (**Figure 3**). The allelic effects of the shared parent are highly dependent on the genomic background of the other parent, and distinct intergenomic interactions in the two populations largely result in the detection of different QTL (Li et al., 2026). In previous studies, two populations shared a common parent Jinmai 919 were used to detect QTL for FLT, however, no same major QTL was identified (Wang et al., 2022; Xu et al., 2024). No common major QTL for the same flag leaf traits (FLL, FLW, FLA and flag leaf angle) was detected in two RIL populations that shared a common parent 20828 (Ma et al., 2020; Tu et al., 2021). These studies further verified that the genetic background has a significant impact on QTL mapping results.

### 4.4 Novel and environmental stable QTLs controlling FLT

In this study, *QFLT.suas-2SY-3B.2*, *QFLT.suas-2CN-4B.2*, *QFLT.suas-2CN-6A* and *QFLT.suas-2SY-6A* were mapped to the intervals of 19.45–22.95 Mb on chromosome 3B, 31.71–34.90 Mb on chromosome 4B, 55.62–66.42 Mb on chromosome 6A, and 54.33–72.74 Mb on chromosome 6A, respectively (**Figures 2**, **3**). There have been relatively few major QTLs for FLT detected in wheat (**Supplementary Table 4**). For example, *QFLT-2B* and *QFLT-6A* were mapped on chromosomes 2B (38.34–66.37 Mb) and 6A (6.62–12.49 Mb), respectively (Wang et al., 2022). The physical and confidence interval of *QFLT-6A* were different from those of *QFLT.suas-2CN-6A* and *QFLT.suas-2SY-6A*. *Qflt-3D.1*, *Qflt-3D.2*, *Qflt-4A* and *Qflt-5A* were located at the peak markers *D1116044-3D16.7* (54.01 Mb) on chromosome 3D, *D7341764-0* (135.23 Mb) on chromosome 3D, *D4539007-4A132* (176.40 Mb) on chromosome 3D, and *D3956865-5A36.6* (61.16 Mb) on chromosome 5A, respectively (Niu et al., 2025). They were different from the chromosomes where the four major QTLs in this study were located. Xu et al. (2024) identified two major QTLs and seven minor QTLs for FLT. *Qflt.saw-3B* was physically mapped to a 65.41–77.48 Mb region on chromosome 3B. Its physical and confidence interval were different from those of *QFLT.suas-2SY-3B.2*. The remaining QTLs were mapped on chromosomes 2B, 4A, 5A, 6B, 7A and 7B, which are also different from the chromosomes where the four major QTLs in this study were located. Therefore, we supposed that the four major QTLs detected in this study were probably novel.

ANOVA revealed that the environment explained the largest proportion of phenotypic variation in both populations (**Table 2**), underscoring the strong influence of external conditions on FLT. Notably, the G×E interaction was also extremely significant (*P* < 0.01), indicating that the QTL effects were not consistent across environments. In the 2CN population, environment was the major factor affecting FLT, while in the 2SY population, both genetic and environmental factors jointly contributed, suggesting population-specific differences in genetic sensitivity to environmental factor. Although FLT was strongly influenced by environments, four major QTLs were identified across multiple environments in this study, confirming that these loci are major and environmental stable QTLs. However, these loci needed further verification using closely linkage marker in near-isogenic lines and other populations. These findings highlight the importance of evaluating QTL stability across environments; therefore, major QTLs consistently detected in multiple environments should be prioritized for further fine mapping and candidate gene validation.

### 4.5 Possible gene interaction between *QFLT.suas-2CN-4B.2* and *QFLT.suas-2CN-6A*

Epistasis, an additive-by-additive interaction between QTL or a nonallelic interaction of homozygous loci, was recognized to describe a situation where the action of one locus masks the allelic effects at another locus (Bocianowski, 2013). It is ubiquitous in cereal crops and serve as a critical component of the genetic basis underlying key agronomic traits (Li et al., 2020; Viana and Souza, 2023; Yu et al., 2002). In this study, we detected two major and stable QTLs for FLT in the 2CN population (**Table 3**). FLT increased significantly by 3.22% when only the positive allele from *QFLT.suas-2CN-4B.2* was present, whereas no statistically significant increase in FLT was observed when only the positive allele from *QFLT.suas-2CN-6A* was present (**Figure 5A**). In addition, the combination of positive alleles of *QFLT.suas-2CN-4B.2* and *QFLT.suas-2CN-6A* significantly increased FLT by 8.28% (**Figure 5A**). Further, no epistatic interaction was detected between *QFLT.suas-2CN-4B.2* and *QFLT.suas-2CN-6A* (**Supplementary Table 5**). Thus, we speculate that there is a possible non-epistatic interaction between *QFLT.suas-2CN-4B.2* and *QFLT.suas-2CN-6A*, and that the positive allele of *QFLT.suas-2CN-4B.2* can mask the effect of *QFLT.suas-2CN-6A.* However, this possible interaction needs to be further confirmed through fine mapping and map-based cloning.

### 4.6 Preliminary prediction of candidate genes for *QFLT.suas-2CN-6A/QFLT.suas-2SY-6A*

In this study, an overlapping physical interval of 10.80 Mb was observed between *QFLT.suas-2CN-6A* and *QFLT.suas-2SY-6A* (**Figures 3A–C**). Therefore, we used this physical interval (55.62–66.42 Mb) to analyze the candidate genes of *QFLT.suas-2CN-6A*/*QFLT.suas-2SY-6A*. In this interval, 115 high confidence and 184 low confidence genes were annotated in the CS v2.1 genome (**Supplementary Table 6**). Of the 115 high confidence genes, expression pattern analyses showed that 34 genes were expressed in leaf and the remaining genes almost not expressed in leaf (**Supplementary Figure 3A**). Further analysis of the expression revealed that 9 genes were significantly higher expression levels compared with other tissues (**Supplementary Figure 3B**). *TraesCS6A03G0192800* encodes phototropic-responsive NPH3 family protein which impacted coleoptile and root phototropism in rice (Haga et al., 2005). *TraesCS6A03G0196600*, which encodes a chloroplast protein (protein low PSII accumulation 3), is a homolog of *LPA3* in *Arabidopsis*, which assists in PSII CP43 assembly (Cai et al., 2010). The remaining seven genes all encode chlorophyll a-b binding protein which increased cell volume, leaf size and number in tobacco (Labate et al., 2004), implying that they potentially affects FLT formation.

Therefore, we speculated that the seven genes (*TraesCS6A03G0215300*, *TraesCS6A03G0216400*, *TraesCS6A03G0216700*, *TraesCS6A03G0216800*, *TraesCS6A03G0216900*, *TraesCS6A03G0217000* and *TraesCS6A03G0217100*) encoding chlorophyll a-b binding protein were likely closely related to FLT in wheat. Nevertheless, these genes represent potential candidates that require further functional validation by expression validation, sequence polymorphism analysis, or haplotype analysis.

Moreover, the 10.80 Mb physical interval remained relatively large and contained 115 high confidence annotated genes. Although expression-based filtering prioritized seven chlorophyll a-b binding protein genes, the current QTL mapping resolution is insufficient to determine the causal genes. Fine mapping with larger populations or near-isogenic lines is required to narrow this interval. Therefore, the proposed candidate genes should be regarded as preliminary candidates requiring further validation rather than as confirmed causal genes.

## 5 Conclusions

In this study, we mapped the QTLs for wheat FLT using two RIL populations in five independent environments. A total of 12 loci were identified, of which four major and stably expressed loci, *QFLT.suas-2CN-4B.2*, *QFLT.suas-2CN-6A*, *QFLT.suas-2SY-3B.2*, and *QFLT.suas-2SY-6A*, were considered as putatively novel. *QFLT.suas-2CN-6A* and *QFLT.suas-2SY-6A* were co-located, and their favorable alleles significantly increased the SNS by 3.21%–3.31%. A co-localized locus (*QFLT.suas-2CN-6A*/*QFLT.suas-2SY-6A*) on chromosome 6A harbors seven candidate genes that encode chlorophyll a-b binding proteins, suggesting their potential involvement in the formation of FLT. These putative candidate genes were identified and provide targets for future fine mapping and functional validation.

## Data Availability

The original contributions presented in the study are included in the article/**Supplementary Material**. Further inquiries can be directed to the corresponding author.
