# Supplementary material for: Novel loci for flag leaf thickness with breeding potential: genetic dissection and candidate genes prediction
Source: Front Plant Sci. 2026 Jul 29;17:1906045. doi: 10.3389/fpls.2026.1906045 (PMC13462394; doi:10.3389/fpls.2026.1906045)
Supplement: Supplementary file 1 [file DataSheet1.docx]

**Supplementary Figure 1** Frequency distribution of flag leaf thickness (FLT) in the 2CN (A) and 2SY (B) populations.

**Supplementary Figure 2** Effects of *QFLT.suas-2CN-6A* (A–C) and *QFLT.suas-2SY-6A* (D–F) on yield traits. * represents significant differences at the 0.05 levels.

**Supplementary Figure 3** Expression analysis of predicted genes in the interval of *QFLT.suas-2CN-6A/QFLT.suas-2SY-6A*. (A) Expression analysis of all genes in this interval; (B) Expression patterns of nine candidate genes for *QFLT.suas-2CN-6A/QFLT.suas-2SY-6A*.
